# In Silico possibilities to understand peri-implant bone healing- state of the Art

**DOI:** 10.1186/s40729-025-00659-x

**Published:** 2025-11-23

**Authors:** Gargi Shankar Nayak, Bilal Al-Nawas

**Affiliations:** 1 Department of Otorhinolaryngology Head and Neck Surgery, Bio-Mechanics & Materials group, University Medical Center, Johannes Gutenberg-University Mainz, 55131 Mainz, Germany; 2 Department of Oral and Maxillofacial Surgery, University Medical Center, Johannes Gutenberg-University, 55131 Mainz, Germany

**Keywords:** Bone healing, Dental implants, Osseointegration, *In-silico* study, Finite element method, artificial intelligence, biomechanics

## Abstract

**Purpose:**

This scoping review was carried out to discover and compare all the possibilities the researchers have thought of in the recent past to perform in silico studies on bone healing after implantation of dental implants.

**Methods:**

An electronic search was conducted in Pubmed, Web of Science, Science Direct and google scholar database to find out related articles in dental peri-implant healing simulations from the period of 2010 until 2025.

**Results:**

In total, 40 articles were found relevant for this review. Different theories have been applied in the literature to simulate the mechanobiology of bone healing. Success has been found in predicting bone healing via in silico studies. The finite element was used often for these studies; however, the application of artificial intelligence is increasing with time in this sector.

**Conclusions:**

In silico platforms provide a non-invasive and fast approach to study the bone healing process. They can be used as an aid to predict peri-implant bone healing in dentistry. The rise of artificial intelligence in this sector opens a new path, where these studies can be performed with high accuracy at an astounding fast pace. These methods can be a boon to clinicians, patients as well as implant developers.

**Supplementary Information:**

The online version contains supplementary material available at 10.1186/s40729-025-00659-x.

## Introduction

 Dental implants are one of the prominent ways to restore the missing tooth function and aesthetics. Thus, they have become an integral component in the field of dentistry. However, there is always a risk of implant failure. The challenge for the dental implant development is in the expansion of understanding of all the aspects, which influences its performance. The success of the dental implant relies on its capability to achieve osseointegration, which relies on the combination of biomechanical and biological factors [1, 2]. Osseointegration is a term coined by Brånemark and his co-workers, which is described as the ability of the implant to form a functional and mechanical contact with the neighbouring osseous tissue with the formation of interpositioned connective tissue [3]. The crucial aspect of osseointegration lies in the healing and remodelling of the host bone site where the implant has been placed via surgery [4]. The healing of these bones takes place through a chain of events which is generally known as secondary bone healing [5, 6]. The healing starts with the blood clot (soft callus) formation and vascularization with the healing gap. Afterwards, mesenchymal stem cells (MSCs) are migrated towards healing callus from surrounding bone marrow, which under *favourable conditions* differentiate and proliferates into osteoblasts, which further forms woven bones, via osteogenesis. The final stage of this process consists of bone remodelling, where the bone is remodelled continuously via osteoblasts and osteoclasts to achieve the rigidity and biomechanical stability of the normal bone based on functionality and mechanical loading [7, 8]. The optimal mechanical environment requirement for these different healing stages differs from each other [9], given to that loading conditions (micro-strains) needs to be modulated throughout the bone healing process. If the loading conditions are *unfavourable*, MSCs can differentiate into soft fibrous tissues that provide minimal mechanical stability and can lead to implant failure [10]. Moreover, even on the bone remodelling stage the amount and direction of loading is crucial, as uneven loading can lead to bone resorption, causing implant failure and increased risk of fracture of the adjacent bones [11, 12]. These loading scenarios can be influenced by a variety of factors such as implant material, implant geometry, surface properties of the implant, occlusal loads, bone density of the patient etc [1–18]. Thus, the study of peri-implant bone healing is important not only for a better patient care, but also for the improvement of implant design, choosing of right materials for implants etc.

In the past, various biological studies have been performed to understand the mechanism behind the peri-implant bone healing phenomenon [19–21]. However, as osseointegration is a continuous biological process involving the ever changing and complex bone remodelling process, it is challenging to investigate it clinically. Moreover, in vivo trials are expensive and involves ethical issues. Thus, in silico platforms to investigate bone healing around dental implants is becoming progressively attractive in this field.

Many theories have been developed in the recent past to have a comprehensive look of the bone healing process. Pauwels was one of the first author to propose tissue differentiation based on mechanical stimulus [22]. He proposed that hydrostatic compressive stress is a specific stimulus for cartilage formation, whereas distortional shear stress is a specific stimulus for fibrous connective tissue formation; bone formation only begins after mechanical stabilization via soft tissues. Along with time, several other bone-healing models have been developed by the researchers [23–28]. A comprehensive review about existing bone healing models can be found in following literature reviews: [29, 30]. However, these models are typically used for modelling long bone fracture healing [31–36]. From time to time, these models have also been used for dental implant applications. Thus, the aim of this review is to find out the latest trends in the recent past for modelling the dental peri-implant bone healing process and to figure out the advantages and disadvantages of these different methods.

## Method

### Focus question

“What in silico possibilities are available for dental peri-implant bone healing/remodelling investigations?”

### Literature search

An electronic search was conducted in Pubmed, Web of Science, Science Direct and google scholar database to find out related articles in dental peri-implant healing simulations. Additional data were also gathered via random and unstructured hand search was also performed based on the experience of the authors.

### Inclusion criteria

Only the articles where bone healing or remodelling was simulated for dental peri-implant bone and were open access were taken in this study. In total, 40 articles were found relevant for the review.

### Exclusion criteria

The exclusion criteria were as follows: non-english publication and duplicated articles. Along with that, studies where FEM simulations were used only for the determination of mechanical conditions were also excluded from the review.

### Screening

**Fig. 1:**
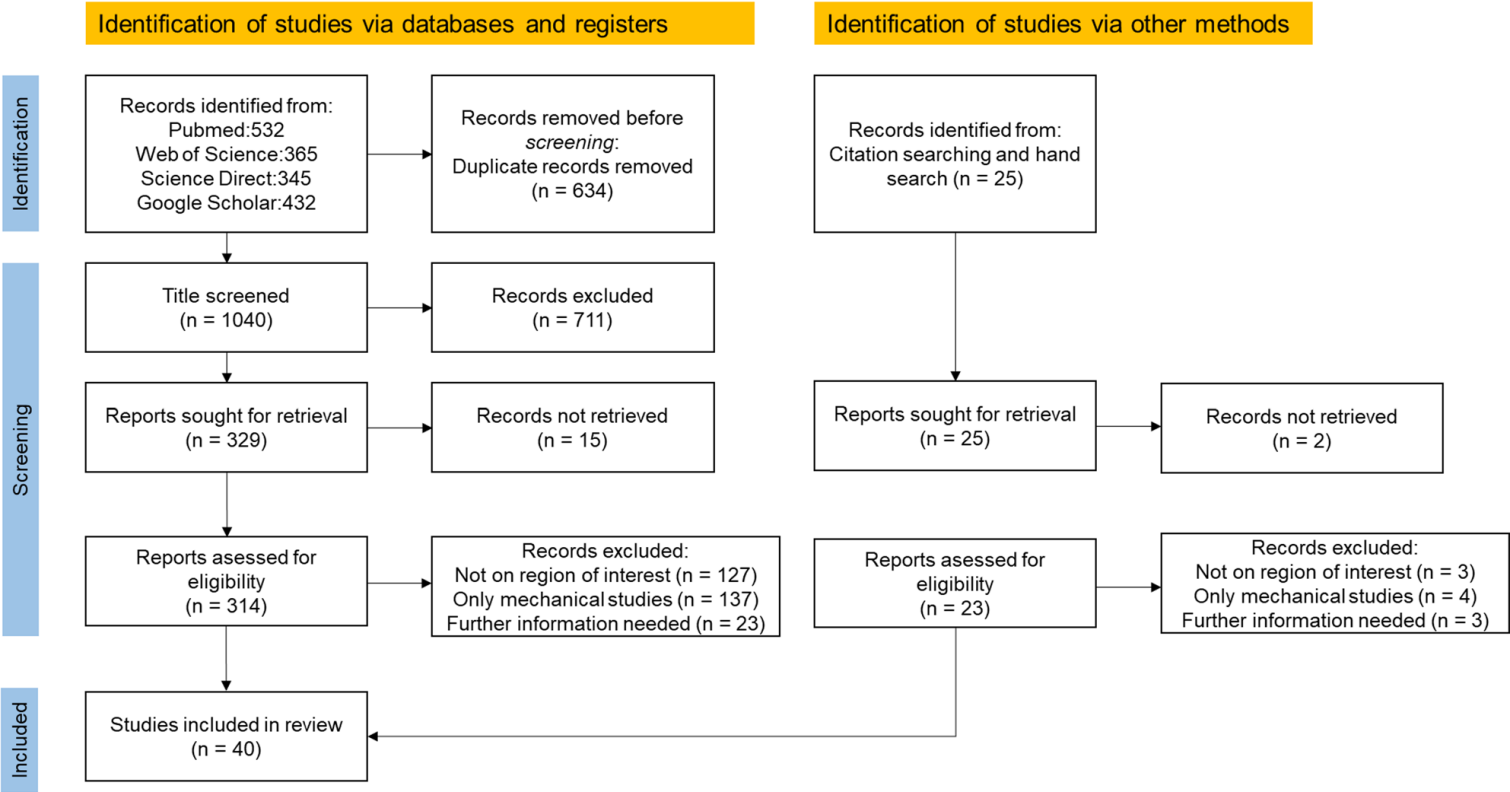
The systematic review flowchart of the study selection process (PRISMA flowchart [37]). N stands for the number of publications

In order to investigate the relevance of focus question in the recent studies we collected the data from 2010 to 2025 and following keywords were used: “dental peri-implant FEM simulation”, “dental peri-implant bone healing simulation”, “dental peri-implant in silico studies”, “dental peri-implant bone remodelling”. As a result, 314 articles were found. Additionally, via, citation searching and hand search 23 more relevant articles were found. The detailed description of the methodology can be found in Fig. 1.

### Data classification

Based on the data, the relevant works have been classified based on the applied methodology: Strain energy density based models, strain based models, tissue-differentiation theory based models, and artificial intelligence based models. To get the detailed list of all the articles taken in this study, see the supplementary material.

## *In silico* possibilities findings

A variety of bone healing/remodelling algorithms have been used in the last 15 years to simulate the dental peri-implant bone healing. These studies generally applied Finite element method (FEM) for their investigations. The models found in the literature has been summarized in Table 1. In the following section, these major algorithm applied in the literature have been discussed.

**Table 1:** Different simulation methodologies applied for bone healing/remodelling simulations in the literature

Applied model	Functioning process	References
Strain energy density based	Bone density change is correlated to strain energy density of the relevant area. Threshold is defined in the model.	[38–51]
Strain based	Bone denstiy change is correlated to micro-strain values of the relevant area. Threshold is defined in the model.	[16–58]
Tissue-differentiation theory based	Differentiation of mesenchymal stem cells into different cell types (fibrous tissue, cartilage, immature bone, mature bone or resorption) is dependent on the combination of different strain conditions.	[6, 7], 59– [63]
Artificial intelligence based	Predictive models are developed by training AI via patient data. AI-based Surrogate models are also developed to expedite the simulation time for bone healing models.	[64–70]

### Strain energy density based models

**Fig. 2:**
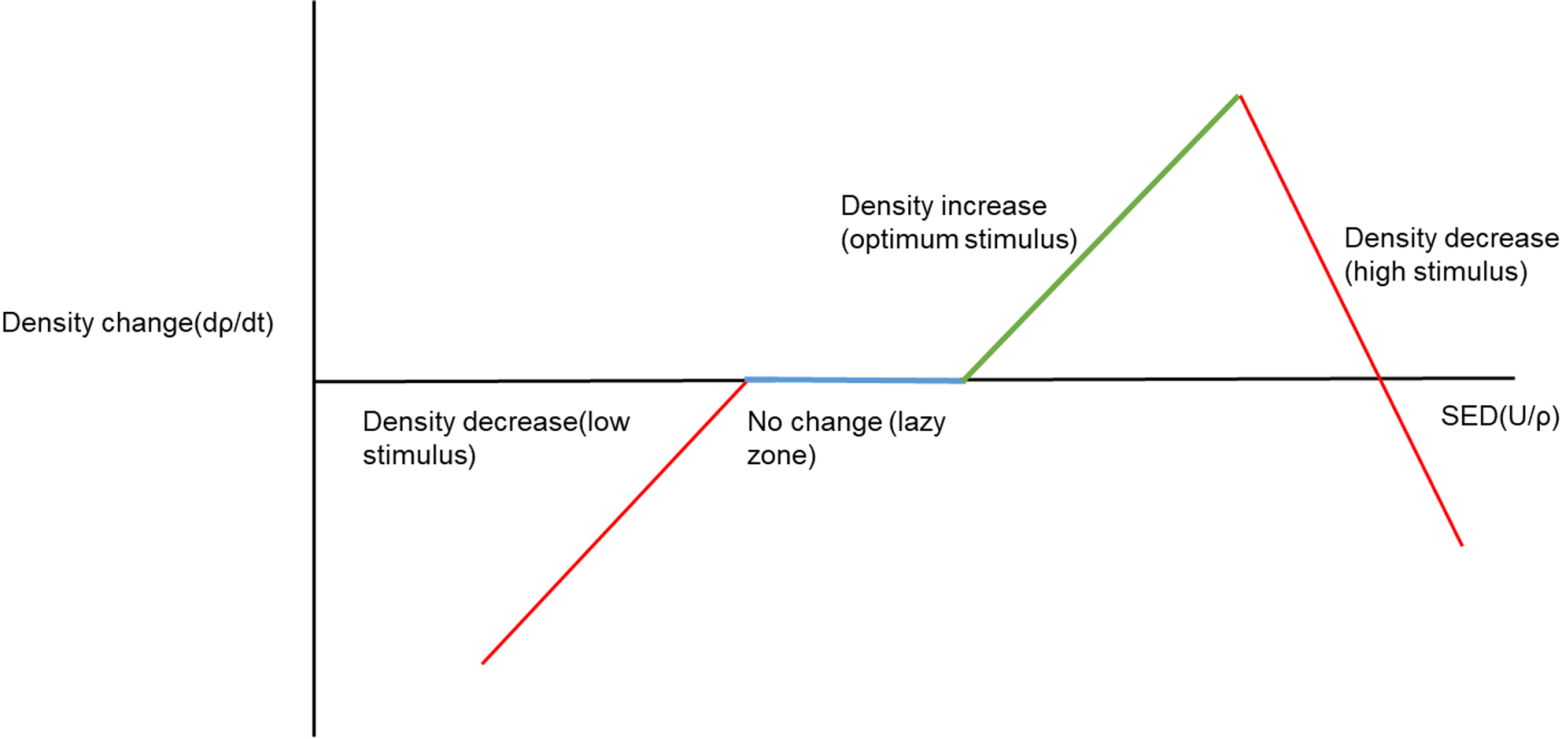
Change in bone density due to SED based mechanical stimulus (ρ is density, t is time and U is strain energy)

Strain energy density (SED) based models are the most sought-after ones in the dental field. The model has been first proposed by Huiskes et al. where SED has been defined as the mechanical stimulus, whose threshold decides increase, decrease or no change in bone density, see Fig. 2 [71]. A variety of equations has been used in the literatures to define the remodelling rate in their studies [38–43]. Generally, the Young’s Modulus of the bone is also related to the density via empirical relationships in these studies.

In a recent study, S. Roy et al. studied the influence of volume fraction (VF) of hydroxyapatite in the bone remodelling potential of Titanium-hydroxyapatite functionally graded material using SED algorithm [42]. To have a realistic model for simulation, they developed a 3D model from the computed tomography (CT) scan data of a 40-year-old individual. Bone remodelling algorithm applied in this study were as follows:

1 $$ \Delta \rho ^{{\left( k \right)}} ~ = \left\{ {\begin{array}{*{20}l} {\omega \frac{{\psi ^{{\left( k \right)}} }}{{\rho ^{{\left( k \right)}} }} - ~\left( {1 + \delta } \right)\Xi ~\Delta t^{{\left( k \right)}} ~,~} & {if~\frac{{\psi ^{{\left( k \right)}} }}{{\rho ^{{\left( k \right)}} }} > \left( {1 + \delta } \right)\Xi } \\ {0~,} & \begin{gathered} ~~if~\left( {1 - \delta } \right)\Xi \le \frac{{\psi ^{{\left( k \right)}} }}{{\rho ^{{\left( k \right)}} }} \hfill \\ \quad \quad \quad \le ~\left( {1 + \delta } \right)\Xi \hfill \\ \end{gathered} \\ {\omega \frac{{\psi ^{{\left( k \right)}} }}{{\rho ^{{\left( k \right)}} }} - ~\left( {1 - \delta } \right)\Xi ~\Delta t^{{\left( k \right)}} ~,} & {if~\frac{{\psi ^{{\left( k \right)}} }}{{\rho ^{{\left( k \right)}} }} < \left( {1 - \delta } \right)\Xi } \\ \end{array} } \right. $$

Where, Δƿ is change in apparent density, *ψ* is SED, ω is remodelling constant and was set to 60 and 120 (month.g/cm^5^), *Ξ* is the remodelling reference value and was set to 0.000036 J/g.cm^− 3^, *Δt* is the duration of the remodelling process, and *δ* is the the extent of lazy zone and was set to 10% in this study *(k)* indicates the k^th^ iteration in the simulation cycle.

Density was updated via Euler’s forward integration algorithm:

2 $$\:{\rho\:}^{(k+1)}={\rho\:}^{\left(k\right)}\:+\varDelta\:{\rho\:}^{\left(k\right)}$$

The changes in density corresponded to change in the Young’s modulus of bone. Here they applied different empirical relationship for cortical and cancellous bone:

3 $$\:{E}_{cortical}=-23.93+24\rho\:$$

4 $$\:{E}_{cancellous}=2.349{\rho\:}^{2.15}$$

They found out that the increase in the VF of Hydroxyapatite in the composite led to a better bone remodelling by decreasing the stress shielding effect and promoting a more uniform stress distribution.

In another study J. Du et al. investigated the potential of SED based algorithms to determine the causes behind bone density decrease after tooth extraction surgeries, via 2D simulations [41]. They applied slightly different bone remodelling algorithm in their work:

5 $$ \rho = \left\{ {\begin{array}{*{20}l} { - 0.05\rho ,} & \begin{gathered} {\text{bone}}\:{\text{loss}}\:{\text{at}} \hfill \\ {\text{constant}}\:{\text{rate,}} \hfill \\ \end{gathered} & {{\text{for}}\:S < S_{l} } \\ {\left( {S - \left( {1 - \delta } \right)S_{0} } \right)B\Delta t,} & {bone\:loss,} & \begin{gathered} {\text{for}}\:S_{l} < S \hfill \\ < (1 - \delta \:)S_{0} \hfill \\ \end{gathered} \\ {0.} & {equilibrium,} & \begin{gathered} {\text{for}}\:\left( {1 - \delta \:} \right)S_{0} < \hfill \\ S < (1 + \delta \:)S_{0} \hfill \\ \end{gathered} \\ {\left( {S - \left( {1 + \delta } \right)S_{0} } \right)B\Delta t,} & {bone\:growth,} & \begin{gathered} {\text{for}}\:\left( {1 + \delta \:} \right) \hfill \\ S_{0} < S < S_{u} \hfill \\ \end{gathered} \\ {0.05\rho ,} & \begin{gathered} bone\:growth\: \hfill \\ at\:constant\:rate, \hfill \\ \end{gathered} & {{\text{for}}\:S > )S_{u} } \\ \end{array} } \right. $$

where *S*_*0*_ is the reference mechanical stimulus (SED per unit bone mass in this case), *S*_*l*_ and *S*_*u*_ are lower and upper limits of mechanical stimulus, *δ* is the half width of the lazy zone, B is the remodelling rate constant and *Δt* is the time step. They related the change in density to Young’s modulus via the single equation given by Carter and Hayes [72]:

6 $$\:E=C{\rho\:}^{3}$$

**Fig. 3:**
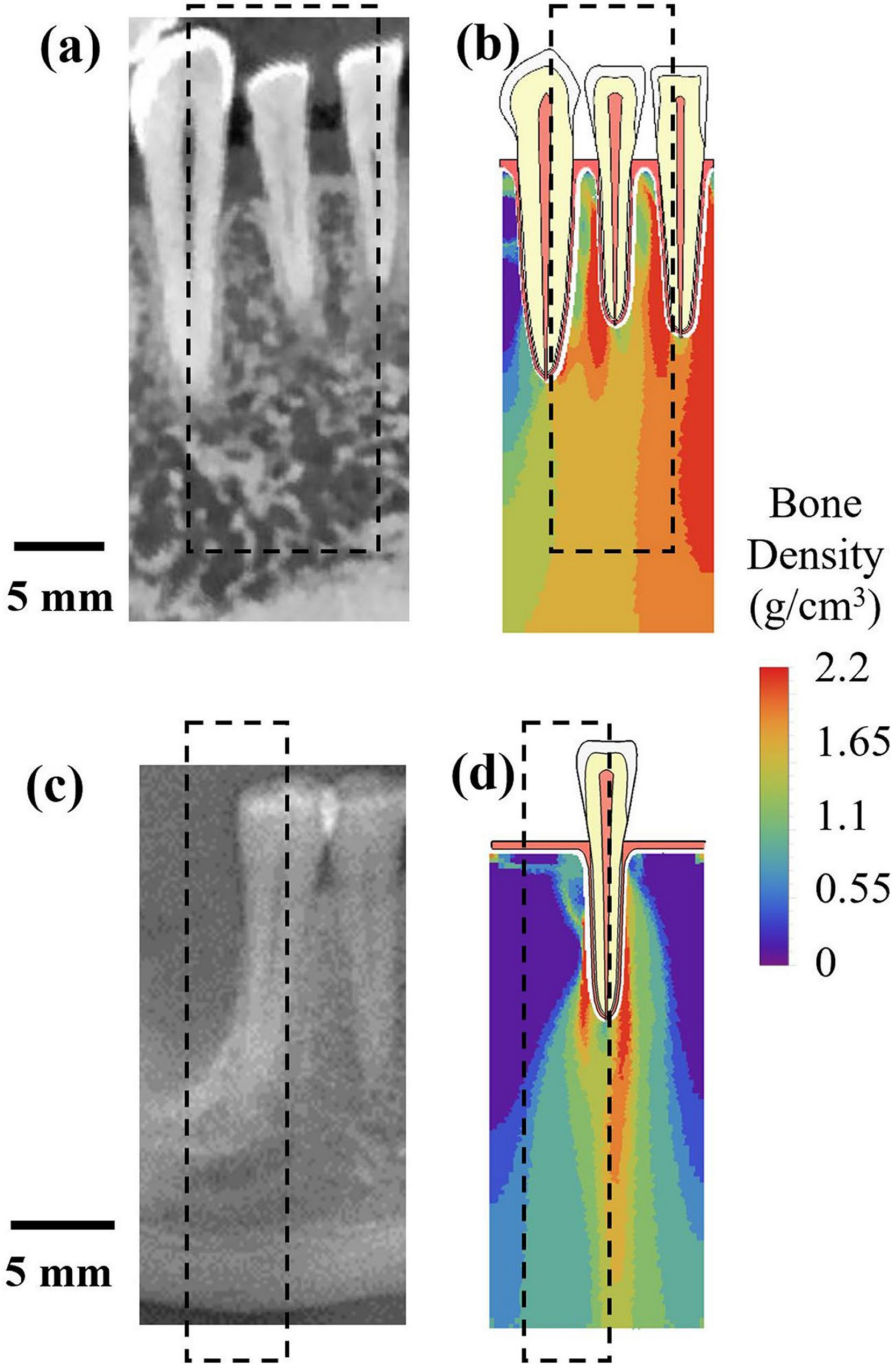
Comparison between CBCT images with computed density distribution via SED algorithm in the work from Du et al. (a) a mesial-distal CBCT image for a region of mandible containing canine, lateral incisor and central incisor; (b) computed density distribution for a 3-tooth model at last iteration step; (c) a mesial-distal CBCT image for a section of mandible with a missing tooth; (d) computed density distribution for a single-tooth model at last iteration step. Similarity in the density distribution in both cases can be seen. It is reprinted from an open access Journal [41]

They compared the change in bone density distribution in their computation, with the cone beam computed tomography (CBCT) in vivo data. The simulation predicted similar density distribution for both healthy and missing tooth scenario, see Fig. 3. Other researchers have performed similar studies, where with the help of SED based models, they were able to predict peri-implant bone remodelling after dental implant placements [8, 38, 39, 46–49] as well as the influence of dental implant design and positioning on bone remodelling [44, 45].

In his doctoral thesis, S. Celik proposed further modification of SED based models for dental implants, where even density change at the early stage of healing can be modelled [51]. He calculated the local change in density using the equation given by J. Li et al. [50]. :

7 $$\:\frac{d\rho\:}{dt}=\left\{\begin{array}{c}0,\:\:if\:S\:\in\:\:\left[\left(1-w\right)k,\left(1+w\right)k\right]\\\:B\left(S-k\right)-D{\left(S-k\right)}^{2},\:\:otherwise\end{array}\right.$$

where *B* and *D* are constants, *S* is SED, *k* is the threshold value of the stimulus, and *w* is the width of the lazy zone. He divided the tissue in several types such as connective tissue, soft callus, intermediate callus and stiff callus. For the remodelling of these different tissue types, different values of *k* and different value range of densities were taken. This study provided a possibility to simulate the different stages of peri-implant bone healing. However, in this study no realistic cases were taken to verify the model. Thus, it is important to validate this model with realistic scenarios to make it accessible for clinical studies.

## Strain based models

Apart from SED based model, strain based model given by H. Frost [25] have also been applied in the dental field [16–58]. Similar to SED based models, in these models strain values are defined as the mechanical stimulus or changes in the bone densities correlate to the strain distribution in the system.

**Fig. 4:**
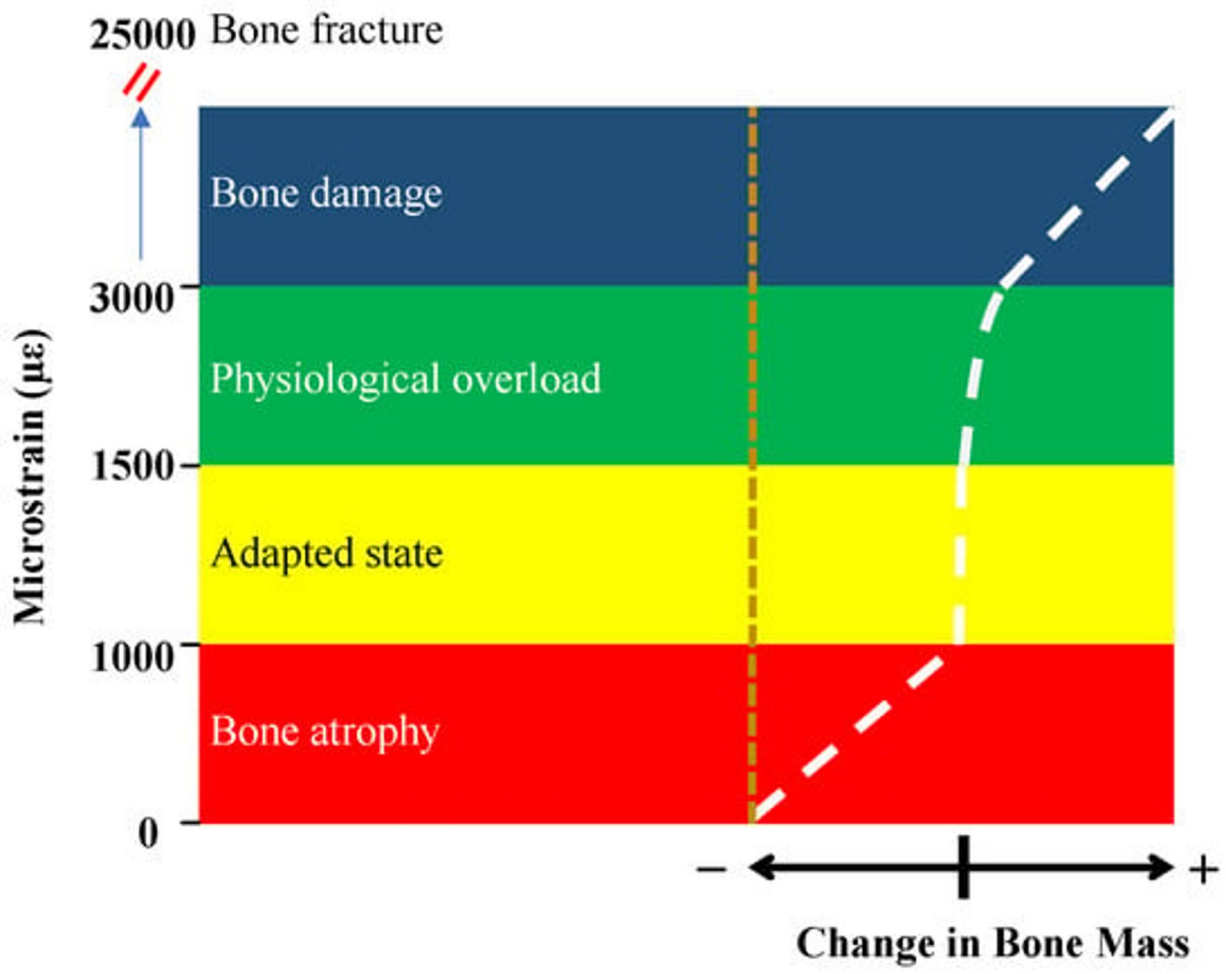
The bone-healing window based on Frost’s mechanostat theory applied in the study by Mehboob et al. It is reprinted from an open access Journal [56]

In a recent study, H. Mehboob et al. applied the Frost’s theory to investigate the impact of porous implants on peri-implant healing [56]. They developed a simplified model of mandible bone, where they calculated the octahedral shear strain distribution in the peri-implant bone for different implant cases under normal chewing force. They took Ti6Al4V bulk implant as control and compared it with the Ti6Al4V implants with different porosities. They took two bone cases in their study: healthy bone and the osteoporotic bone. In addition, two scenarios were taken: full osseointegration, where bone and implant are in total contact and partial osseointegration, where relative contact with bone and implants are allowed. The healing window applied in the study can be seen in Fig. 4. It was found out that the porosities improved the strain distribution range for healthy bone in partial and full osseointegration cases for better bone healing. However, for osteoporotic bone it was seen that all the implants led to conditions where bone damage is highly possible. Thus, this works gives the insights that even though porous implants can improve the bone healing, in the case of osteoporotic bones biting forces also needs to be managed, so that the patients do not suffer from implant failures.

## Tissue-differentiation theory based models

**Fig. 5:**
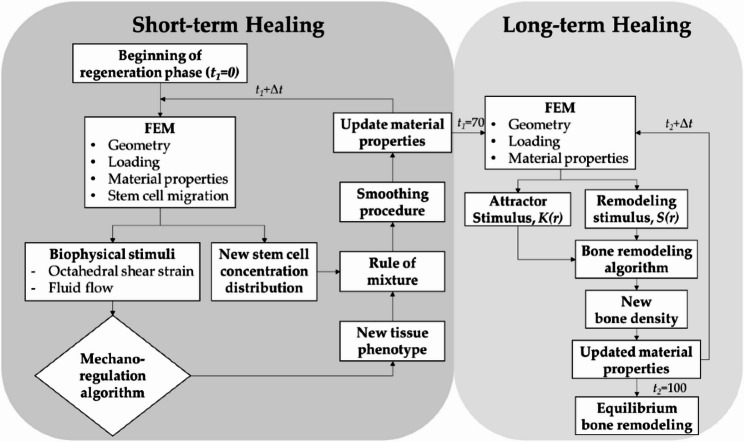
The bone-healing algorithm applied in the study by Kung et al. It is reprinted from an open access journal [63]

Apart from density based theories, tissue differentiation theories for bone healing such as the one given by L. Claes et al. [23] and the poroelastic theory given by P. Prendergast et al. [24] have also been applied in dentistry [6, 7, 59–63]. These theories predict the differentiation of mesenchymal stem cells into different tissue types such as fibrous tissue, cartilage, immature bone, mature bone or resorption/destruction based on the biomechanical conditions. In a recent study, A. Kung et al. combined the poroelastic theory for bone healing and SED based bone remodelling theory to predict the long-term peri-implant bone healing [63]. The modelling algorithm applied for this hybrid model can be seen in Fig. 5. This model showed good correlation with the results found in the experimental studies. Thus, tissue differentiation theory based models carry the potential to have an in-depth analysis of the early stage bone healing process.

### Artificial intelligence based models

With the advancement in artificial intelligence (AI), its application has been increasing at a fast pace in dentistry [73]. In the recent years, several works have been done to predict the peri-implant bone condition at different stages via deep learning network (DLN) based models [64–70]. J. Cha et al. successfully developed a convolutional neural network (CNN) to predict the peri-implant bone loss using periapical radiograph images [67]. A region-based CNN was trained using 708 periapical radiograph images (508 for training, 100 for validation and 100 for testing), where the key points at the interface between bone and implant were chosen as the region of interest for training. Using the detected keypoints on the radiographic images, the CNN models determined the extent of bone loss. Interestingly, the level of detection of CNN reached the level of diagnosis given by the experts.

In another interesting work, P Kung et al. developed a DLN to predict the peri-implant bone healing under different dental implants, patient’s age and gender, and occlusal forces [70]. Initially, the bone healing simulation results were obtained via FEM model, where the poroelastic theory based bone healing model was applied. These results were fed to DLN for training. Afterwards DLN was tested as a surrogate for the FE calculation. DLN was able to predict the results that can be obtained in FEM with 97.23% accuracy. Moreover, the computation time with DLN was significantly shorter than that of FEM. Where, FEM model took an hour to predict tissue differentiation throughout 35 days, DLN needed only 50 s to achieve that result. This study showed the high potential AI holds to provide insights on peri-implant bone healing prediction, which can expedite the implant development as well as assist the dental implant treatment.

**Fig. 6:**
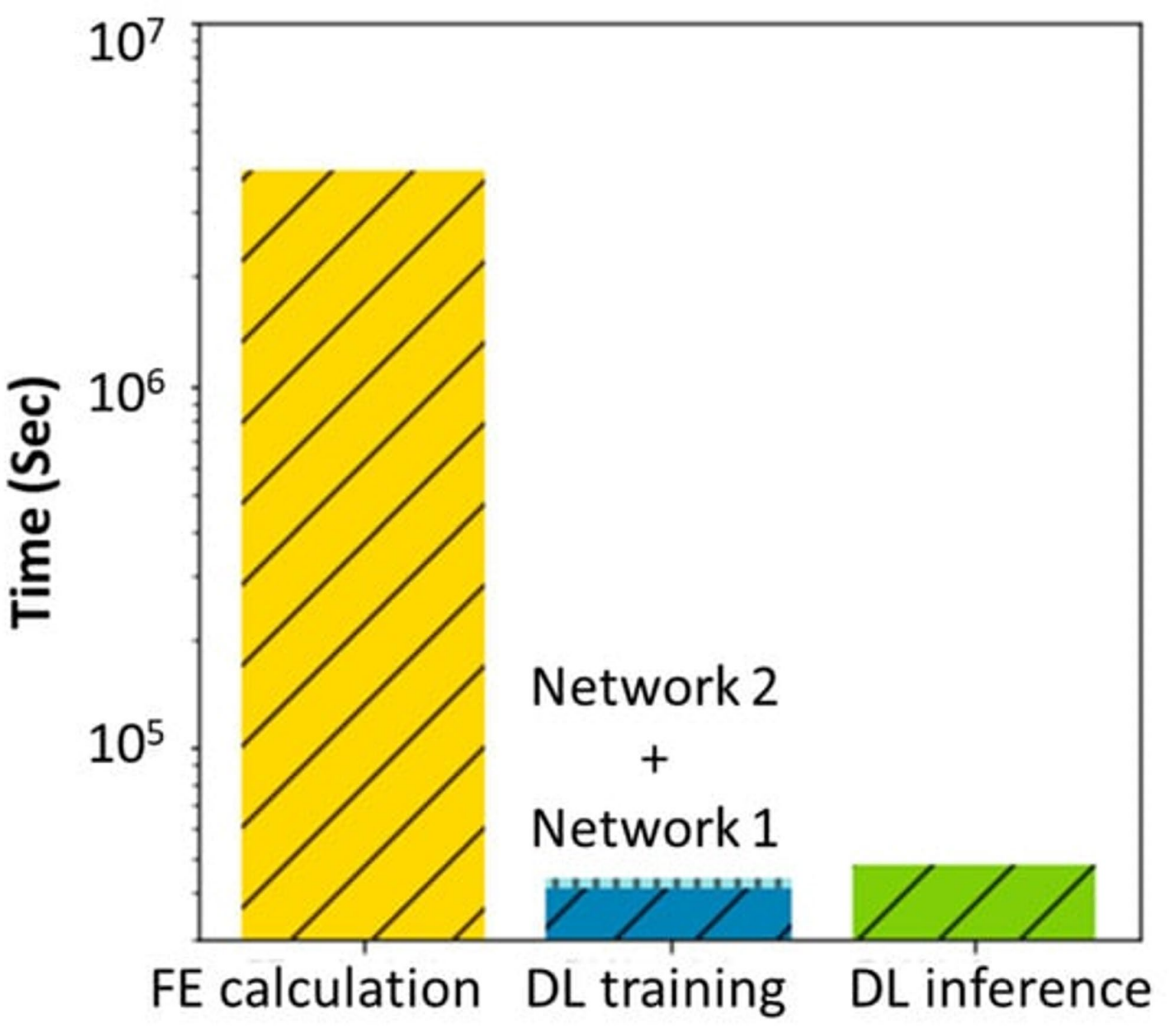
The computational time needed to calculate tissue differentiation for 900 cases by FEM vs. DLN in the study by Kong et al. It is reprinted from an open access journal [70]

## Discussion

Bone healing is a complicated phenomenon, which depends on a multitude of factors. To replicate bone healing in in silico environment, various methodologies can be applied. This could be phenomenological such as strain based or SED based models. In such models the apparent density of bone is correlated with empirical equations, which are intelligently developed by observing the real healing scenarios. The researchers have been able to quantitatively verify the validity of these models with the real patient data. These models are predominantly used to measure bone remodelling. However, in recent years, the equations are also developed to measure the density change in the early stage of bone healing for the dental field. The major challenge in such models is the determination of empirical values in the equation. These values differ in various literatures; thus, a consensus needs to be established for the successful application of such models in the dental field.

On the other hand, tissue-differentiation theories applied mechanobiology for bone healing modelling. The proliferation and differentiation of mesenchymal cells into various tissue types are based on the mechanical conditions. These models have been successfully used in various literatures for bone healing simulations. The major challenge for such models is the accurate determination of right mechanical conditions for tissue differentiation. The validation of the values applied in the literature for tissue differentiation is challenging to perform in experimental conditions. Moreover, the major tissue differentiation theories given by Claes [23]and Prendergast [24] provide different mechanical conditions for bone healing. Thus, these models need to be compared and validated for different clinical scenarios to accurately determine the optimal mechanobiological conditions for dental bone healing.

The application of AI has shown great improvement in the further development of bone healing models. On one hand, AI can help in the prediction of peri-implant bone loss via radiographic images and on the other hand, they can also be trained to be used as surrogate models to perform bone healing simulations at a much faster speed than that of conventional FEM techniques. Such methods can be of great help for the clinicians, not only in patient care but also for the determination of accurate implant strategy. However, AI needs to be further improved and properly trained with experienced professionals for the betterment in this field.

## Conclusion

This review illustrated the various in silico possibilities to simulate the peri-implant bone healing process. The SED based models have been the most popular method found in the literature to simulate the density change of the bone with time. However, for the initial stages of bone healing, tissue-differentiation theory based models have been most often used. The comparison of these techniques with clinical data have been done in the literature, where good predictability has been shown. With the further development of AI, it has also been used in this sector recently. It has shown great promise in the literature to replace the FEM based model, given to its fast and accurate prediction. Overall, in silico studies provide great insights on the peri-implant bone healing conditions, which needs to be readily applied in the dental implant development to expedite the process along with providing fresh perspectives for designing the implants. For clinicians, these models can aid in providing accurate treatment to patients, especially in cases where the risk of implant failure is high.

## Supplementary Information

Below is the link to the electronic supplementary material.

Supplementary Material 1

## Data Availability

No datasets were generated or analysed during the current study.

## Abbreviations

AI: Artificial Intelligence
CBCT: Cone Beam Computed Tomography
CNN: Convolutional Neural Network
CT: Computed Tomography
DLN: Deep Learning Network
FEM: Finite Element Method
MSCs: Mesenschymal Stem Cells
SED: Strain Energy Density
VF: Volume Fraction
