# Supplementary material for: In Silico possibilities to understand peri-implant bone healing- state of the Art
Source: Int J Implant Dent. 2025 Nov 23;11:69. doi: 10.1186/s40729-025-00659-x (PMC12669444; doi:10.1186/s40729-025-00659-x)
Supplement: Supplementary file 1 — Supplementary Material 1 [file 40729_2025_659_MOESM1_ESM.pdf]

Articles taken in review

SED Based studies

1. Park S, Park J, Kang I, Lee H, Noh G. Effects of assessing the bone remodeling process in biomechanical finite element stability evaluations of dental implants. Comput Methods Programs Biomed. 2022 Jun 1;221
2. Li W, Lin D, Rungsiyakull C, Zhou S, Swain M, Li Q. Finite element based bone remodeling and resonance frequency analysis for osseointegration assessment of dental implants. Finite Elements in Analysis and Design. 2011 Aug;47(8):898–905.
3. Lin D, Li Q, Li W, Duckmanton N, Swain M. Mandibular bone remodeling induced by dental implant. J Biomech. 2010 Jan 19;43(2):287–93.
4. Lin CL, Lin YH, Chang SH. Multi-factorial analysis of variables influencing the bone loss of an implant placed in the maxilla: Prediction using FEA and SED bone remodeling algorithm. J Biomech. 2010 Mar 3;43(4):644–51.
5. Su K, Yuan L, Yang J, Du J. Numerical Simulation of Mandible Bone Remodeling under Tooth Loading: A Parametric Study. Sci Rep. 2019 Dec 1;9(1).
6. Rajaeirad M, Fakharifar A, Posti MHZ, Khorsandi M, Watts DC, Elraggal A, et al. Evaluating the effect of functionally graded materials on bone remodeling around dental implants. Dental Materials. 2024 May 1;40(5):858–68.
7. Bourauel C, Celik S, Keilig L, Hasan I. Bone remodelling around dental implants based on functional loading. In: BSSM 13th International Conference on advances in Experimental Mechanics [Internet]. Southampton, United Kingdom; 2018. Available from: https://www.researchgate.net/publication/327423361
8. Assoratgoon I, Wan B, Tenkumo T, Sato T, Kawata T, Putra RH, et al. Three-dimensional in vivo and finite element analyses of peri-implant bone remodeling after superstructure placement.
9. Wang C, Fu G, Deng F. Difference of natural teeth and implant-supported restoration: A comparison of bone remodeling simulations. J Dent Sci. 2015 Jun 1;10(2):190–200.
10. Chou HY, Muftu S. Peri-implant Bone Remodeling Around an Extraction Socket: Predictions of Bone Maintenance by Finite Element Method [Internet]. 2012. Available from: https://www.researchgate.net/publication/230590517
11. Hernandez-Rodriguez Y, Lekszycki T. Finite memory model of bone healing in analysis of moving interface between mandible tissue and bone substitute material after tooth implant application. Continuum Mechanics and Thermodynamics. 2023 May 1;35(3):991–8.
12. Wang C, Li Q, Mcclean C, Fan Y. Numerical simulation of dental bone remodeling induced by implant-supported fixed partial denture with or without cantilever extension. Int J Numer Method Biomed Eng. 2013 Oct;29(10):1134–47.
13. Hasan I, Mit A, Der Mathematisch-Naturwissenschaftlichen G. Computational Simulation of Trabecular Bone Distribution around Dental Implants and the Influence of Abutment Design on the Bone Reaction for Implant-Supported Fixed Prosthesis. 2011.
14. Rungsiyakull C, Chen J, Rungsiyakull P, Li W, Swain M, Li Q. Bone's responses to different designs of implant-supported fixed partial dentures. Biomech Model Mechanobiol. 2015 Apr 1;14(2):403–11.
15. Salih C. Simulation of Bone Remodeling Process around Dental Implant During the Healing Period [Internet]. [Bonn]; 2021. Available from: https://www.researchgate.net/publication/364658341
16. Li J, Li H, Shi L, Fok ASL, Ucer C, Devlin H, et al. A mathematical model for simulating the bone remodeling process under mechanical stimulus. Dental Materials. 2007 Sep;23(9):1073–8.

Strain based models

1. Marcián P, Wolff J, Horáčková L, Kaiser J, Zikmund T, Borák L. Micro finite element analysis of dental implants under different loading conditions. Comput Biol Med. 2018 May 1;96:157–65.
2. Mehboob H, Mehboob A, Abbassi F, Ahmad F, Samad Khan A, Miran S. Bioinspired porous dental implants using the concept of 3D printing to investigate the effect of implant type and porosity on patient's bone condition. 2021; Available from: https://www.tandfonline.com/doi/epub/10.1080/15376494.2021.1971347?needAccess=true
3. Eser A, Tonuk E, Akca K, Dard MM, Cehreli MC. Predicting bone remodeling around tissue- and bone-level dental implants used in reduced bone width. J Biomech. 2013 Sep 3;46(13):2250–7.
4. Field C, Li Q, Li W, Thompson M, Swain M. A comparative mechanical and bone remodelling study of all-ceramic posterior inlay and onlay fixed partial dentures. J Dent. 2012 Jan;40(1):48–56.
5. Lee HC, Tsai PI, Huang CC, Chen SY, Chao CG, Tsou NT. Numerical Method for the Design of Healing Chamber in Additive-Manufactured Dental Implants. Biomed Res Int. 2017;2017.
6. Martinello PA, Cartagena-Molina AF, Capelletti LK, Fernandes BV, Franco APG de O, Mercuri EGF, et al. Adding mechanobiological cell features to finite element analysis of an immediately loaded dental implant. Eur J Oral Sci. 2024 Aug 1;132(4).
7. Mehboob H, Ouldyerou A, Ijaz MF. Biomechanical Investigation of Patient-Specific Porous Dental Implants: A Finite Element Study. Applied Sciences (MDPI). 2023 Jun 1;13(12).
8. Mehboob H, Mehboob A, Abbassi F, Ahmad F, Khan AS, Miran S. Bioinspired porous dental implants using the concept of 3D printing to investigate the effect of implant type and porosity on patient's bone condition. Mechanics of Advanced Materials and Structures. 2022;29(27):6011–25.
9. Ouldyerou A, Aminallah L, Merdji A, Mehboob A, Mehboob H. Finite element analyses of porous dental implant designs based on 3D printing concept to evaluate biomechanical behaviors of healthy and osteoporotic bones. Mechanics of Advanced Materials and Structures. 2022;30(11):2328–40.

Tissue differentiation theory based models

1. Chou HY, Müftü S. Simulation of peri-implant bone healing due to immediate loading in dental implant treatments. J Biomech. 2013 Mar 15;46(5):871–8.
2. Irandoust S, Müftü S. The interplay between bone healing and remodeling around dental implants. Sci Rep. 2020 Dec 1;10(1).
3. Babayi M, Ashtiani MN, Emamian A, Ramezanpour H, Yousefi H, Mahdavi M. Peri-implant cell differentiation in delayed and immediately-loaded dental implant: A mechanobiological simulation. Arch Oral Biol. 2023 Jul 1;151.
4. Rousseau N, Chabrand P, Destainville A, Richart O, Milan JL. Mechanobiological model to study the influence of screw design and surface treatment on osseointegration. Comput Methods Biomech Biomed Engin. 2022;25(3):273–89.
5. Smolin A, Eremina G, Martyshina I, Xie J. Biomechanics of Osseointegration of a Dental Implant in the Mandible Under Shock Wave Therapy: In Silico Study. Materials. 2024 Dec 1;17(24).
6. Li MJ, Kung PC, Chang YW, Tsou NT. Healing pattern analysis for dental implants using the mechano-regulatory tissue differentiation model. Int J Mol Sci. 2020 Dec 1;21(23):1–13.
7. Kung PC, Chien SS, Tsou NT. A hybrid model for predicting bone healing around dental implants. Materials. 2020 Jun 2;13(12):1–15.

Artificial intelligence based models

1. Huang N, Liu P, Yan Y, Xu L, Huang Y, Fu G, et al. Predicting the risk of dental implant loss using deep learning. J Clin Periodontol. 2022 Sep 1;49(9):872–83.
2. Lyakhov PA, Dolgalev AA, Lyakhova UA, Muraev AA, Zolotayev KE, Semerikov DY. Neural network system for analyzing statistical factors of patients for predicting the survival of dental implants. Front Neuroinform. 2022 Dec 7;16.
3. Huang N, Liu P, Yan Y, Xu L, Huang Y, Fu G, et al. Predicting the risk of dental implant loss using deep learning. J Clin Periodontol. 2022 Sep 1;49(9):872–83.
4. Çelik B, Çelik ME. Automated detection of dental restorations using deep learning on panoramic radiographs. Dentomaxillofacial Radiology. 2022;51(8).
5. Cha JY, Yoon HI, Yeo IS, Huh KH, Han JS. Peri-implant bone loss measurement using a region-based convolutional neural network on dental periapical radiographs. J Clin Med. 2021 Mar 1;10(5):1–12.
6. Oh S, Kim YJ, Kim J, Jung JH, Lim HJ, Kim BC, et al. Deep learning-based prediction of osseointegration for dental implant using plain radiography. BMC Oral Health. 2023 Dec 1;23(1).
7. Zhang C, Fan L, Zhang S, Zhao J, Gu Y. Deep learning based dental implant failure prediction from periapical and panoramic films. Quant Imaging Med Surg. 2023 Feb 1;13(2):935–45.
8. Kung PC, Hsu CW, Yang AC, Chen NY, Tsou NT. Prediction of Bone Healing around Dental Implants in Various Boundary Conditions by Deep Learning Network. Int J Mol Sci. 2023 Feb 1;24(3).
